# Biochemical Assessment and Monitoring of Mitochondrial Disease

**DOI:** 10.3390/jcm7040066

**Published:** 2018-03-29

**Authors:** Iain P. Hargreaves

**Affiliations:** 1 Department of Pharmacy and Biomolecular Science, Liverpool John Moores University, Byrom Street, Liverpool L3 5UA, UK; i.hargreaves@ucl.ac.uk; Tel.: +44-151-231-2711; 2 Neurometabolic Unit, National Hospital, Queen Square, London WC1N 3BG, UK

Mitochondrial respiratory chain (MRC) disorders have a multifaceted clinical presentation and genetic origin. The adage, “any symptom, any organ or tissue, any age of presentation, any mode of inheritance”, coined by Munnich and colleagues in 1992 [1] highlights the challenges faced in diagnosing these complex disorders, which requires a multidisciplinary approach involving the results of the clinical, histological, genetic, and biochemical investigations. In the biochemical context, the first-line investigations to determine evidence of a MRC disorder in patients are by the assessment of plasma or cerebral spinal fluid (CSF) lactate levels [2]. However, these determinations lack specificity and sensitivity, and a ‘normal’ result does not exclude the possibility of an underlying MRC disorder. An elevated plasma alanine level, an indicator of cellular pyruvate accumulation, has also been suggested as an appropriate marker of MRC dysfunction with an absolute level >450 μM being utilized as factor to determine the likelihood of mitochondrial disease, according to the Nijmegen diagnostic protocol [3]. However, an elevated plasma alanine level may only be present during a relapse in symptoms, and therefore a ‘normal’ plasma alanine level does not exclude an underlying MRC disorder [2]. Urine organic acid analysis may reveal evidence of elevated lactate, Krebs cycle intermediates, or 3-methylglutaconic acid in some patients with MRC disorders; however, these metabolites may only be present if the patient is acutely symptomatic and be absent during periods of stability [2]. However, the diagnostic utility of urine organic acid analysis in mitochondrial disease is supported by the study of Alban et al. (2017) [4], which reported an abnormal urine organic acid profile in 82% of patients with muscle MRC enzyme deficiencies. Nonetheless, renal immaturity is an important factor to consider, and an abnormal urine organic acid profile in a patient less than one year of age should be interpreted with extreme caution [2].

The diagnosis of mitochondrial disease is impeded by the paucity of reliable surrogate markers of MRC dysfunction presently available to select in preference to an invasive skeletal muscle biopsy, which is required for spectrophotometric enzyme assay. However, the hormone-like cytokine, serum fibroblast growth factor-21 (FGF-21), which is involved in the intermediary metabolism of carbohydrates and lipids, has been suggested as a potential reliable biomarker of MRC dysfunction [5]. In addition, the growth differentiation factor-15 (GDF-15) has also been identified as a potential marker of mitochondrial disease [6]. Although, at present, there are still concerns about the sensitivity of FGF-21 for detecting MRC disease in non-myopathic patients, and GDF-15 is regarded to have superior sensitivity but lower specificity [7]. A study by Morovat et al. [8] has indicated that although serum FGF-21 determination may have diagnostic utility in mitochondrial disease, it may prove more useful in monitoring disease progression and the effects of therapeutic intervention. Furthermore, the combined use of serum FGF-21 determination with urine organic acid analysis has also been suggested to improve the diagnostic value of either test used in isolation [4]. Surprisingly, however, the combined assessment of both FGF-21 and GDF-15 in adult patients with mitochondrial disease was not found to improve the diagnostic value of the individual tests [7]. An elevated plasma creatine level has also recently been suggested as a potential biomarker of mitochondrial disease; however, in view of the number of variables that may influence the circulatory level of this compound, its diagnostic value requires careful consideration [9].

The determination of coenzyme Q_10_ (CoQ_10_) in plasma or blood mononuclear cells (BMNCs) appears to be of diagnostic utility in identifying patients with a deficit in the level of thisisoprenoid [10]. However, although this determination can’t distinguish between primary or secondary CoQ_10_ deficiencies, it identifies an important subset of mitochondrial patients that may respond to CoQ_10_ supplementation [11]. The use of BMNC’s also offers a means to directly assess MRC enzyme activities in patients with suspected mitochondrial disease, although a ‘normal’ result does not exclude the possibility that a defect may be expressed in other tissues [12].

The assessment of oxidative stress is also an important consideration in the context of mitochondria disease; although not a diagnostic parameter, it can provide important information about disease pathophysiology as well as the therapeutic efficacy of antioxidant strategies. The intracellular redox status of the antioxidant, reduced glutathione (GSH), as indicated by the ratio of GSH to its fully oxidised form, GSSG in white blood cells or BMNCs, may offer an appropriate surrogate for this evaluation [13].

Overall, at present, due to the lack of reliable validated biomarkers or surrogates for evaluating evidence of MRC dysfunction [14], spectrophotometric assessment of MRC enzyme activities in a skeletal muscle biopsy or tissue from the disease-presenting organ if accessible is still considered the ‘Gold Standard’ biochemical method for diagnosing patients with MRC disorders. The status quo is set to exist until more effort and funding can be centered on identifying appropriate biomarkers that fulfill all criteria required to have diagnostic utility for detecting MRC disorders.
